# Monocular blur alters the tuning characteristics of stereopsis for spatial frequency and size

**DOI:** 10.1098/rsos.160273

**Published:** 2016-09-21

**Authors:** Roger W. Li, Kayee So, Thomas H. Wu, Ashley P. Craven, Truyet T. Tran, Kevin M. Gustafson, Dennis M. Levi

**Affiliations:** 1 School of Optometry, University of California, Berkeley, CA 94720, USA; 2 Helen Wills Neuroscience Institute, University of California, Berkeley, CA 94720, USA

**Keywords:** stereoacuity, visual acuity, coarse and fine stereopsis, optimal observer, anisometropic amblyopia

## Abstract

Our sense of depth perception is mediated by spatial filters at different scales in the visual brain; low spatial frequency channels provide the basis for coarse stereopsis, whereas high spatial frequency channels provide for fine stereopsis. It is well established that monocular blurring of vision results in decreased stereoacuity. However, previous studies have used tests that are broadband in their spatial frequency content. It is not yet entirely clear how the processing of stereopsis in different spatial frequency channels is altered in response to binocular input imbalance. Here, we applied a new stereoacuity test based on narrow-band Gabor stimuli. By manipulating the carrier spatial frequency, we were able to reveal the spatial frequency tuning of stereopsis, spanning from coarse to fine, under blurred conditions. Our findings show that increasing monocular blur elevates stereoacuity thresholds ‘selectively’ at high spatial frequencies, gradually shifting the optimum frequency to lower spatial frequencies. Surprisingly, stereopsis for low frequency targets was only mildly affected even with an acuity difference of eight lines on a standard letter chart. Furthermore, we examined the effect of monocular blur on the size tuning function of stereopsis. The clinical implications of these findings are discussed.

## 1. Introduction

Stereopsis, based on binocular disparity (i.e. the differences between the two retinal images), adds a third dimension of space to the visual world [1–3]. Underlying the detection of binocular disparity is a bank of spatial filters of different scales and orientations in the visual brain. In this conceptual framework, low spatial frequency channels provide the basis for coarse stereopsis while high spatial frequency channels provide for fine stereopsis [4,5]. Retinal disparity is first detected by binocular simple cells in the primary visual cortex [6–10]. There are two types of operating units: phase and position disparity detectors [11–13] with monocular Gabor-like two-dimensional receptive fields in each eye. In order to better understand the neural network sub-serving stereopsis, a great deal of research has been done to characterize the functional properties of these basic operators [14–18].

We are particularly interested in learning how the processing of stereopsis is altered in response to an imbalance in binocular input. In clinical situations, it is not uncommon to see patients present with unequal visual acuity in the two eyes [19,20]. For example, in the case of uncorrected anisometropia (i.e. the two eyes have unequal refractive errors) [21], the optical image formed on the retina is more blurred in one eye than the other, resulting in an unequal reduction in visual acuity and contrast sensitivity. It is important to note that visual blur affects contrast sensitivity to different extents for low and high spatial frequency stimuli [22–25].

It is well documented that monocular blurring of vision reduces stereoacuity [26–38]. However, previous studies have used tests that are broadband in their spatial frequency content. Characterizing the effect of monocular stimulus blur on stereoacuity performance for different spatial frequencies, from coarse to fine [39], is important for a better understanding of the underlying mechanisms. In this study, we developed a new stereoacuity test based on narrow-band Gabor stimuli [40] in order to quantify the effect of monocular blur on stereoacuity for different spatial frequency stimuli. More importantly, we revealed the spatial frequency tuning of stereopsis under a wide range of blur conditions. In addition to the carrier spatial frequency, Gabor targets contain another visual cue, the Gaussian envelope. Using a modified stimulus paradigm involving Gaussian blobs, we also studied how monocular blur influences the envelope size tuning of stereopsis. Our experiments provide new insights into how the visual system, as a multiple channel analyser, processes blurred visual inputs for stereoscopic vision.

## 2. Material and methods

### 2.1. Observers

Our experiments included altogether 10 young university students, 20–25 years of age. All had normal or corrected-to-normal visual acuity of 20/16^−2^ or better in each eye, with the interocular acuity difference of two letters or less on a standard LogMAR letter chart (National Vision Research Institute of Australia, 1978). Inclusion criteria were spherical refractive error in the range of +0.25 to −5.00 D and astigmatism in the range of 0 to 0.75 D. None of the participants had anisometropia of greater than 0.50 D (spherical equivalent). All participants had normal stereoacuity of 20 arcsec or better (Randot® stereotest, Stereo Optical Co., Inc., Chicago, IL, USA).

### 2.2. Visual stimuli

**Figure 1.**
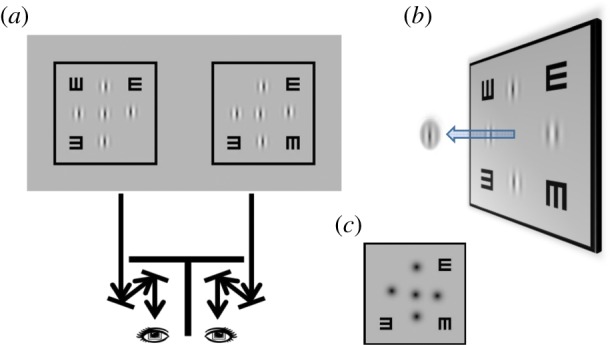
Experimental set-up. (*a*) The visual stimulus consisted of two horizontally separated square blocks, one presented to each eye. Each block contained a Gabor target patch surrounded by four Gabor reference patches. Stereo disparity was introduced by shifting the two Gabor targets in opposite directions. A haploscope was used to enable binocular fusion. (*b*) Cyclopean perception. The visual task was to determine the stereoscopic depth of the Gabor target (crossed disparity: in front; uncrossed disparity: behind) relative to the four references. (*c*) A variant of our stereoacuity test was used in the second experiment using Gaussian blobs.

The visual stimulus consisted of two horizontally separated black squares. At the centre of each square, there was a target Gabor (or Gaussian) patch surrounded by four reference Gabor (or Gaussian) patches (figure 1*a*). A custom-built four-mirror haploscope was used to present a half monitor screen to each eye (i.e. the left square to the left eye and the right square to the right eye). Binocular disparity was introduced by shifting the two target patches, one in each block, in opposite horizontal directions. In order to eliminate any potential monocular cues, the position and carrier phase of each target and reference Gabor patch were randomly jittered based on a uniform distribution (vertical and horizontal position range, ±200–1600 arcsec—more than an order of magnitude larger than the observers' stereo thresholds; phase range, 0–360^o^). All visual stimuli were displayed on a 21-inch Sony F520 flat monitor screen at 1800 × 1440 resolution and 90 Hz refresh rate. The mean luminance of the stimuli was 55 cd m^−2^ and the contrast of each Gabor patch was 99%.

### 2.3. Cyclopean view

Figure 1*b* illustrates the cyclopean percept of the visual stimuli in binocular viewing. The square frame served as a fusion lock to achieve proper eye alignment. The two ‘*E*’s presented binocularly at the top-right and bottom-left corners served as an accommodation lock for bringing the visual stimulus in focus. The ‘*E*’s presented monocularly at the top-left (only seen by the left eye) and bottom-right corners (only seen by the right eye) served as a binocular status indicator ensuring the absence of monocular suppression during testing, and proper alignment (when perceived to be one above the other). The visual task was to determine the stereoscopic depth of the target Gabor (in front or behind) relative to the four adjacent reference Gabor patches. Trial-by-trial audio feedback was provided for each response.

### 2.4. Psychophysical methods

For each trial, the amount of binocular disparity between the target Gabors presented to each eye was determined by two interleaved adaptive staircases to track the threshold: one was for crossed disparity (the target patch appears in front of the reference patches), while the other was for uncrossed disparity (the target patch appears behind the reference patches). The trials were divided into triplets: three correct responses decreased the disparity magnitude by one unit step, two correct responses left the disparity unchanged, and only one or no correct response increased the disparity by two unit steps. The starting disparity was roughly two times the individual observer's threshold disparity, and the step size was roughly one-third to half of the threshold disparity. Stereoacuity was defined as the disparity at the 84% correct response rates (*d*′=1) obtained by fitting a Probit function. Each run consisted of 160 response trials: 75 trials of crossed disparity, 75 trials of uncrossed disparity and 10 trials with zero disparity.

### 2.5. General experimental design

Bangerter foils (Ryser Optik, St Gallen, Switzerland), available in a range of densities (0.1–1.0), were used to reduce visual acuity in the dominant eye. These foils act like a Gaussian filter in reducing contrast monotonically with increasing spatial frequency [41]. Stereo thresholds were measured for a range of interocular acuity difference: from 0 to 8 lines on a LogMAR letter chart, with a total of six blur levels: D0, 1, 2, 4, 6 and 8 (e.g. D8: non-dominant eye, LogMAR −0.1 and dominant eye, LogMAR 0.7, *D*_LogMAR_ = 0.7−[−0.1] = 0.8). Note that the acuity differences reported were based on our clinical measurements, not the filter designation provided by the manufacturer.

In the first experiment, we investigated the influence of induced monocular blur on the spatial frequency tuning of stereopsis. The carrier spatial frequency of the Gabor patch varied from 1.25 to 20 cpd (V1, 2, 5, 7, 10, 15 and 20, where the number denotes the spatial frequency in cycles/degree: 1.25, 2.5, 5, 7.5, 10, 15 and 20 cpd, respectively). The standard deviation of the Gaussian envelope determined the stimulus size. Envelope size, or stimulus size, was defined as one standard deviation (*σ*) ranging from 1.75 to 28 arcmin (V1 [28′], 2, 5, 7, 10, 15 and 20 [1.75′]). The envelope size of the Gabor patch was inversely proportional to carrier frequency, keeping a constant number of grating cycles/standard deviation. In the second experiment, we employed a modified stimulus design, in which all the Gabor patches were replaced by Gaussian blobs (figure 1*c*), in order to explore how monocular blurring alters the size tuning characteristics of stereopsis. Note that Gn (G1–20) has the same envelope size as Vn (V1–20).

### 2.6. Stimulus scaling

The stimulus spatial scale was manipulated by adjusting the physical size on the screen and varying the viewing distance: 50 cm (V1 and G1), 1 m (V2 and G2) or 2 m (V5–20 and G5–20). For V5 (or G5) stimuli viewed at 2 m, the envelope size was 7 arcmin, the reference-target centre-to-centre distance was 48 arcmin when positional jittering was disabled, the ‘*E*’ size was 25 × 25 arcmin and the square frame was 197.3 arcmin. The inter-pixel distance was 20 arcsec at a viewing distance of 2 m (50 cm, 80 arcsec); sub-pixel accuracy was achieved by contrast manipulation.

### 2.7. Procedures

**Figure 2.**
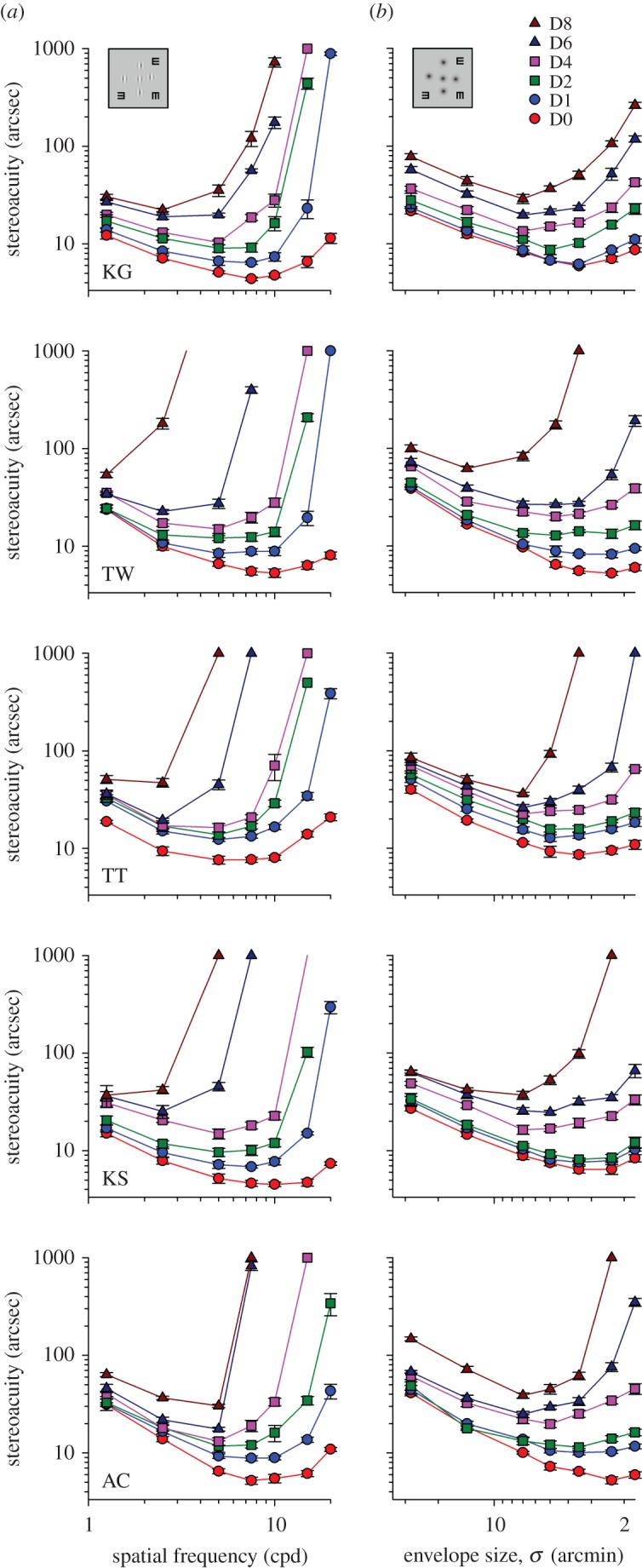
Interocular acuity difference and stereoacuity. (*a*) The effect of induced monocular blur on stereoacuity for Gabor stimuli of different spatial frequencies, from left to right: V1, V2, V5, V7, V10, V15 and V20. (*b*) The effect of induced monocular blur on stereoacuity for Gaussian blobs of different sizes, from left to right: G1, G2, G5, G7, G10, G15 and G20. Bangerter foils were used to induce optical blur in the dominant eye. The figure legend shows the levels of interocular visual acuity difference, Dn, where n denotes the number of lines on a standard LogMAR letter chart (e.g. D8: an 8-line, LogMAR 0.8, difference between eyes). Each individual observer's data is displayed on a separate panel. In the right column, the direction of *x*-axis for envelope size is reversed. Error bars represent the standard errors of the mean.

All testing was done with the observer wearing best optical correction. The condition sequences (blur × frequency × size) were randomized and largely counter-balanced across sessions. Each stereoacuity data point reported in figure 2 represents an average of five to eight threshold measurements. All participants were well experienced in psychophysical testing.

## 3. Results

### 3.1. Spatial frequency tuning of stereopsis

The stereoacuity versus spatial frequency function is a U-shaped curve, with the optimum spatial frequency at approximately 7.5–10 cycles per degree (figure 2*a*, D0: red lines). Using narrow-band stimuli, we clearly showed that optical blur affects low frequency stimuli and high frequency stimuli differently. The raw data of individual observers is illustrated in figure 2*a* (left column, Gabor stimuli; right column, Gaussian blob); note that in each plot both axes are logarithmic. In general, stereo thresholds for the frequency range tested were progressively elevated as interocular acuity difference increased from D1 through D8 (two-way RANOVA, *F* = 8.116, *p* < 0.001), and the effect of optical degradation was more pronounced for high spatial frequency stimuli. There was a significant interaction between the two factors: spatial frequency and acuity difference (two-way RANOVA [V1–V15; D0–D2], *F* = 7.156, *p* < 0.001). Importantly, stereopsis for low frequency targets was only minimally affected, even with an acuity difference of as much as eight lines on a standard LogMAR chart (e.g. V1: a factor of 2.3 in stereoacuity versus a factor of 6.3 in minimum angle of resolution, MAR).

**Figure 3.**
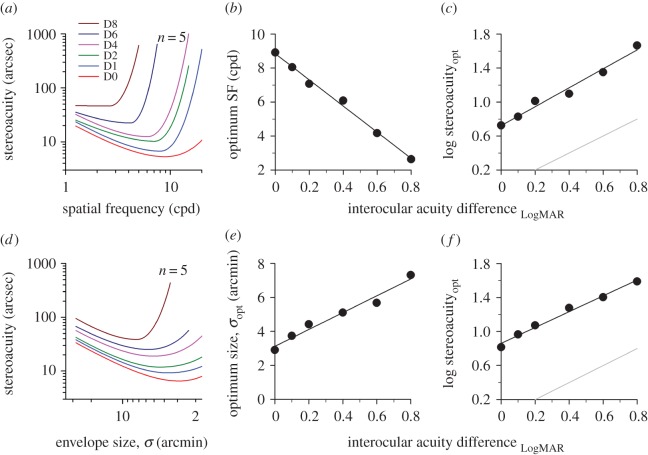
Tuning characteristics of stereopsis. (*a*) Stereoacuity versus spatial frequency function for Gabor stimuli. At each blur level, stereoacuity datasets from all five observers in figure 2 were grouped and fitted with a double Cauchy function. The *x* (optimum spatial frequency, optimum SF) and *y* (stereoacuity at the optimum SF, stereoacuity_opt_) coordinates of the inflection point on each curve are summarized as a function of interocular acuity difference in (*b*) and (*c*), respectively. (*d*) Stereoacuity versus envelope size function for Gaussian blobs. The *x* (optimum envelope size, *σ*_opt_) and *y* (stereoacuity at the *σ*_opt_, stereoacuity_opt_) coordinates of the inflection point on each curve are summarized as a function of interocular acuity difference in (*e*) and (*f*), respectively. Note that the data points are not displayed in (*a*) and (*d*). A grey unity reference line is shown in (*c*) and (*f*) for comparison.

For each blur setting, the data from all five observers were pooled and fitted with a double Cauchy function [42] (figure 3*a*; for clarity the raw data points are not shown). Across all blur levels, the ‘stereoacuity versus spatial frequency’ curve and its turning point (minima) gradually shift up and to the left. Increasing the interocular acuity difference degraded stereoacuity more substantially at high spatial frequencies, gradually shifting the optimum frequency to lower spatial frequencies. The frequency tuning operates linearly in the range of blur levels tested (figure 3*b*, *y* = 7.71*x* + 8.85, *R*^2^ = 0.99; D0: 8.9 cpd; D8: 2.6 cpd). The cut-off acuity difference can be estimated by extrapolating the regression line to where optimum spatial frequency reaches zero. Stereopsis is expected to begin collapsing, or to be dramatically degraded when visual acuity difference between the two eyes increases to LogMAR 1.15, roughly 11–12 lines on a LogMAR letter chart. The sensitivity at the optimum frequency, after logarithmic transformation, degraded linearly with increasing interocular acuity difference (figure 3*c*, *y* = 1.11*x* + 0.72, *R*^2^ = 0.98; D0: 5.3 arcsec; D8: 46.2 arcsec). It should be noted that the slope of the regression line is nearly parallel to the grey unity line (slope difference = 0.11 ± 0.0853, *t* = 1.34, *p* = 0.25; mean vertical distance between two lines = 0.76 ± s.e. 0.03 log unit), meaning that the rate of decrease in stereoacuity was approximately the same as the rate of decrease in monocular visual acuity. Thus, the loss in visual acuity due to optical blur was not amplified in the neural processing of stereo disparity along the visual pathways.

**Figure 4.**
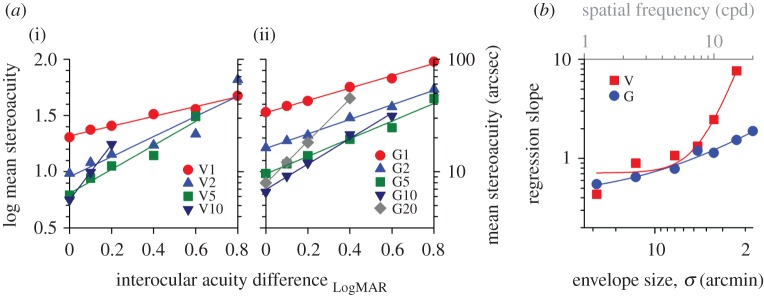
Frequency/size specific losses of stereoacuity. (*a*) Mean stereoacuity as a function of interocular visual acuity difference for different spatial frequencies (V series: Gabor stimuli) (i) and stimulus sizes (G series: Gaussian blobs) (ii). The datasets were fitted with a linear regression model, and the regression slopes are summarized in (*b*) as a function of envelope size. The top grey axis shows the peak spatial frequency for Gabor stimuli. The regression lines for V7 and V15 and G7 and G15 are omitted for the purpose of clarity.

In order to quantify the effect of monocular blur on different spatial frequency channels, mean stereo thresholds are presented as a function of interocular acuity difference in figure 4*a*(i). In some moderate and severe blur settings, no measurable stereoacuity could be obtained, especially at high spatial frequencies, in some (or all) observers. Those conditions were therefore not included in this part of the regression analysis. All the data points shown in the figure represent mean threshold data computed from all five observers. A linear regression model provided a reasonable fit to the data (*R*^2^ > 0.87 for V1–15). On the log–log scale, increasing monocular blur resulted in a linear increase in stereo threshold, with the regression slope becoming steeper as spatial frequency increases. At a given blur level, stereoacuity was more affected and deteriorated much faster at high frequencies than low frequencies. For example, at the blur level of D2 (*D*_LogMAR_ 0.2), stereoacuity for V10 stimuli was degraded by as much as 0.5 log unit (a factor of 3.11). By contrast, stereoacuity was degraded only 0.1 log unit (a factor of 1.26) for V1 stimuli. Note that the datasets of V7 and V15 are omitted in the figure for clarity, but the slope values can be found in figure 4*b* (red symbols; the top axis displays spatial frequency).

### 3.2. Size tuning of stereopsis

The Gabor stimuli presented to our observers were comprised of two visual components: the cue provided by the Gabor carrier (the feature cue) and the cue defined by the Gaussian envelope (the envelope cue). A logical question would be, ‘which cue did human observers actually use in processing our stereo task?’ To answer that question, it is necessary to quantify the response characteristics of the two visual cues in stereo processing. In the second part of the experiment, the same five observers repeated the procedures with a modified stimulus design in which all Gabor patches were replaced with Gaussian blobs. Their threshold data at each blur level are plotted as a function of envelope size in figure 2*b*.

The data from individual observers were binned and then fitted with a double Cauchy function (figure 3*d*, note that the direction of *x*-axis for envelope size is reversed here and in the following figures unless otherwise stated). The general trend of ‘stereoacuity versus envelope size’ function is similar to that of ‘stereoacuity versus spatial frequency’ function. Increasing monocular blur elevated stereo thresholds more for small targets than large targets (e.g. G10 [*σ* = 3.5 arcmin]: a factor of 1.82 versus G1 [*σ* = 28 arcmin]: a factor of only 1.26 at the level of D2); there was a significant interaction between envelope size and interocular acuity difference (two-way RANOVA [G1–G20; D0–D4], *F* = 17.726, *p* < 0.001). The turning point gradually shifted to the left (i.e. larger stimulus sizes; figure 3*e*, *y* = 5*x* + 3.12) and up (figure 3*f*, *y* = 0.93*x* + 0.86) across blur levels. The log-transformed mean stereoacuity correlated well with interocular acuity difference using a linear regression model (figure 4*a*(ii), *R*^2^ > 0.95 for G1–G20). The regression slope was found to increase smoothly with decreasing envelope size from G1 through G20 (summarized in figure 4*b*, blue symbols), indicating that the degradation effect of blurring on stereoacuity had a greater impact on smaller targets. For clarity, the datasets of G7 and G15 are omitted in figure 4*a*(ii), but their slope magnitudes can be found in figure 4*b* (where *σ* = 4.67 and 2.33 arcmin, respectively).

**Figure 5.**
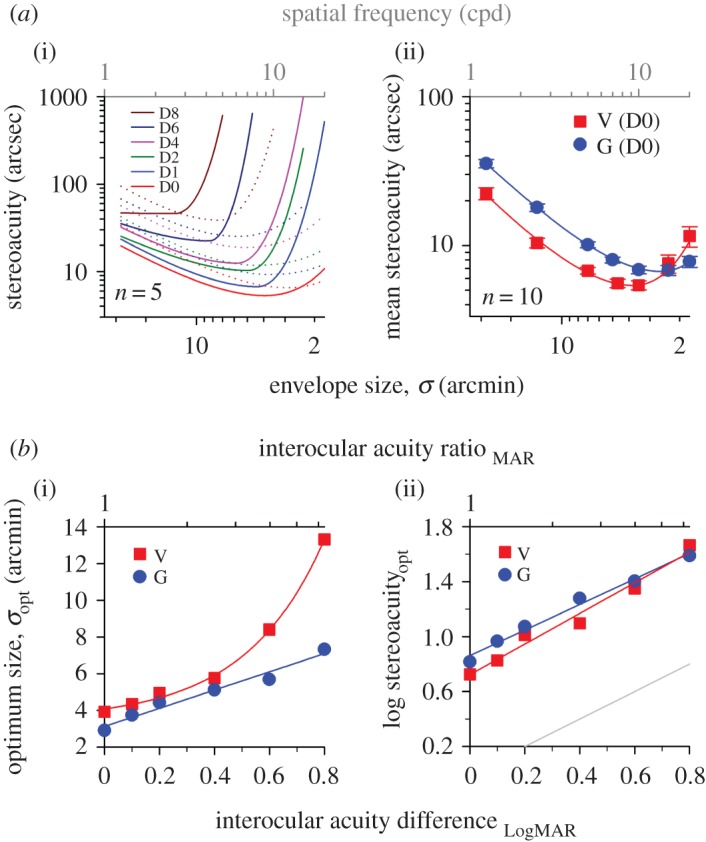
Frequency cue versus size cue. (*a*)(i)The threshold data from figure 3*a*,*d* are replotted and summarized as a function of envelope size. Solid curves: Gabor stimuli. Dotted curves: Gaussian blobs. Colour coding: same as in figure 3*a*,*d*. The top grey axis shows the peak spatial frequency for Gabor stimuli. (ii) A comparison of stereo thresholds for the stimuli comprising Gabor or Gaussian stimuli with zero interocular difference; five more experienced observers, totalling 10 observers were tested in this graph. Error bars represent the standard errors of the mean. (*b*) The difference in stimulus size tuning characteristics of stereopsis for the two types of visual stimuli with monocular blur. (i) Optimum envelope size. (ii) Stereoacuity at the optimal frequency or size.

The stereo thresholds for both Gabor and Gaussian stimuli are superimposed in figure 5*a*(i) (V: solid lines; G: dotted lines; the same colour coding as in figure 3*a*). To facilitate the comparison, stereoacuity was plotted against envelope size, with the top grey axis displaying spatial frequency for Gabor stimuli. At the level of D0, there was a significant interaction between stimulus type and envelope size. For clarity, the data are replotted on an enlarged ordinate scale in figure 5*a*(ii) (two-way RANOVA, *F* = 42.468, *p* < 0.001; five more new experienced observers with normal vision were tested in this plot). The stereothresholds were significantly lower for V1–V7 stimuli than G1–G7 stimuli (the leftmost four data points of each curve: *t* > 2.713, *p* < 0.009), but higher for V20 than G20 (the rightmost data point of each curve: *t* = 4.217, *p* < 0.001). No significant difference in thresholds was found between V10–V15 and G10–G15. As noted above, increasing monocular blur elevated stereo thresholds for the two stereo tests. It is evident that Gabor stimuli were affected much more than Gaussian stimuli, especially when the target size was small. The rates of threshold elevation started to deviate from each other when envelope size was smaller than 4.67 arcmin (figure 4*b*). There were some discrepancies in the tuning characteristics for envelope size between V and G stimuli (figure 5*b*). (i) When interocular acuity difference was larger than LogMAR 0.4, the optimum size was much bigger for V stimuli than G stimuli (figure 5*b*(i); note that the data from figure 3*b,e* are replotted here as a function of envelope size). (ii) As shown in figure 5*b*(ii), our observers seem to have better stereoacuity for V stimuli than G stimuli at the optimum size for a range of acuity difference up to roughly LogMAR 0.4.

## 4. Discussion

We applied a new stereo test based on Gabor features. By manipulating the carrier spatial frequency, we were able to evaluate the effects of monocular blur on individual spatial channels selectively, spanning from coarse to fine stereopsis. Our experiments reveal that disparity processing is affected differently at different spatial scales. Coarse stereopsis that extracts the overall three-dimensional shape information is quite resistant to the visual blur induced by Bangerter filters, whereas fine stereopsis that detects disparity for fine spatial details is much more dramatically impacted. These findings are not too surprising as the Bangerter foil mostly attenuates the middle and particularly high spatial frequency content (roughly quantified by visual acuity), but largely spares low spatial frequencies [41]. As monocular blur increases, the optimum frequency where the most efficient performance is obtained gradually shifts to lower spatial frequencies. When dealing with complex natural visual scenes containing different spatial frequency components (i.e. broadband visual stimuli), it is likely that performance is limited by the higher spatial frequencies in the image. Indeed, previous studies speculate that with higher spatial frequencies being removed, visual blur might shift stereo processing to lower spatial frequencies causing an elevation of threshold [43]. It is worthwhile noting that our participants maintained relatively ‘good’ coarse stereopsis for low spatial frequency stimuli under severely blurred conditions. Our regression analysis provides an estimate of cut-off acuity difference where stereopsis starts collapsing.

Stereoacuity depends not only on stimulus spatial frequency, but also on stimulus size. In a separate experiment using Gaussian blob stimuli, we examined how the stereoacuity versus envelope size function is altered in response to monocular blur and contrasted its differential effects for the two stimulus configurations. For small stimulus sizes, thresholds for the Gaussian task appeared to be relatively more stable at a given blur level (figure 5*a*(i): D0–D4, *σ* < roughly 7 arcmin) when compared with those for the Gabor task. For screening purposes in clinical settings, the Gaussian task might have some advantages over the Gabor task of being less influenced by optical blur when vision is not fully corrected. In turn, this Gaussian blob stereo test is very similar to some natural viewing tasks. For example, judging relative distances of light sources such as street lamps and car headlights in a dark environment, although the luminance polarity is opposite.

**Figure 6.**
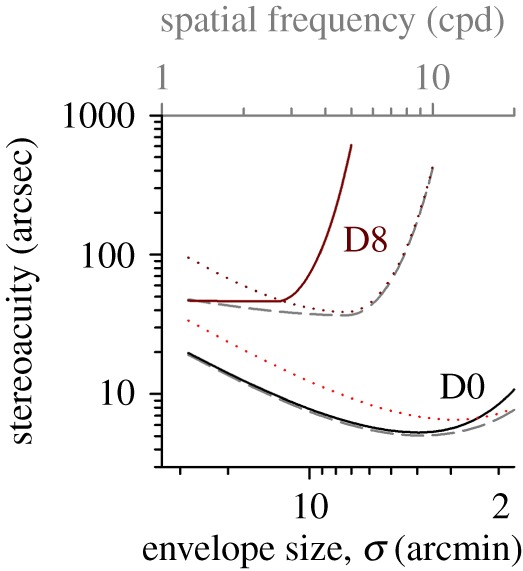
Human observer versus optimal observer. A hypothetical optimal observer was modelled to have access to both spatial frequency and envelope cues and be able to switch to the appropriate visual mechanism possessing better stereo performance (grey dashed lines). The stereoacuity data obtained from the five human observers are replotted in this figure (solid lines: Gabor stimuli; dotted lines: Gaussian blobs). Two monocular blur levels are depicted to contrast the differences in stereoacuity performance between human and optimal observers. For clarity, the curves of the optimal observer are shifted down by 5% in threshold.

There is a fundamental question of how the observers process the two visual components, i.e. carrier and envelope, embedded in our Gabor stereo task. To illustrate this point, a hypothetical optimal observer was modelled to have access to both carrier spatial frequency and Gaussian envelope information of the visual stimuli, and to be able to select the most appropriate visual cue that carried the finest disparity information available for depth processing. As elaborated in the figure, the optimal observer (figure 6, grey dashed lines) used the spatial frequency mechanism possessing lower thresholds when processing larger stimuli up to the intersection point where the solid curve (frequency cue, refer to the top axis; data replotted from figure 3*a*) and dotted curve (envelope cue; data replotted from figure 3*d*) met and thereafter, switched to the envelope cue which in turn offered lower thresholds for smaller stimuli. For clarity purposes, the curves for the optimal observer were shifted down slightly by 5% in threshold. Apparently, there were some discrepancies in performance between human (black solid lines) and optimal observers. After the point of intersection, our observers did not seem to make use of the envelope cue even when it could potentially provide better stereoacuity performance. Perhaps, the visual brain is not capable of reconstructing complete envelope information from our Gabor stimulus configurations. However, the envelope cue does appear to influence stereo perception for Gabor stimuli with more grating cycles [44,45] and other spatial vision tasks [46].

Our stereoacuity test might have potential clinical applications in providing a comprehensive assessment of stereo vision, from coarse to fine stereopsis. For example, this technique could be particularly useful in obtaining a complete picture of stereoacuity performance when prescribing monovision correction for presbyopia [47,48], and also for evaluating patients with monocular vision loss such as cataracts. Ocular diseases usually affect low, medium and high spatial frequencies to a different extent [49–52]. Anisometropic amblyopes often maintain stereopsis at low spatial frequencies, but not at high [53,54]. Under blur conditions, stereopsis for high spatial frequencies collapses, but low spatial frequencies were largely unaffected. Our current findings have clinical implications for understanding both the sparing of coarse stereopsis and the deficits in fine stereopsis in anisometropic amblyopia[55–57]. However, one should be cautious when generalizing our findings to uncorrected anisometropia as Bangerter filters produce diffusive blur that is qualitatively different from defocus blur, notably phase alternation with optical defocus [41]. For defocus blur, the amplitude of the modulation transfer function (MTF) falls below zero and then rises again, damping around zero, for higher spatial frequencies causing spurious resolution [58]. By contrast, the MTF with Bangerter filters does not fall below zero. In addition, the spatial frequency spectra could be dramatically different between the two anisometropic eyes. But that is not the case in our studies, the bandwidth difference is substantially removed with the narrow bandwidth stimuli. Our findings might, in some sense, reflect how interocular contrast differences affect stereoacuity [33]. We are currently investigating the effects of binocular blur versus monocular defocus blur on stereoacuity at different spatial scales.

In summary, we undertook a systematic investigation of how optical blur influences stereo vision at different spatial scales. Using narrow-band visual stimuli, we reported the spatial frequency tuning of stereopsis under blurred conditions. These findings contribute to understanding the operational and functional properties of the basic neuronal mechanisms mediating the sense of depth perception.

## Data accessibility

Data files relevant to this study can be found in the Dryad database: http://dx.doi.org/10.5061/dryad.qf768 [59].
